# Imagined intergroup contact and power-dependent attentional bias: a dual-process model of transnational policy cognition in the belt and road initiative context

**DOI:** 10.3389/fpsyg.2025.1611261

**Published:** 2025-11-21

**Authors:** Xinyu Cheng, Peipei Kang

**Affiliations:** 1 School of Journalism and Communication, Shandong University, Jinan, China; 2 School of Journalism and Communication, Beijing Institute of Graphic Communication, Beijing, China

**Keywords:** policy communication, cross-scale interaction, transnational cognition, imagined intergroup contact, semantic networks, attentional bias

## Abstract

**Introduction:**

With the increasing complexity of transnational policy communication in digital media environments, understanding the cognitive mechanisms behind public perception has become crucial. Although the international community widely recognizes cognition as the foundation for shared knowledge and the construction of global identities, research on the psychological mechanisms and the impact of cognition based on transnational perspectives remains scarce. This paper takes the Belt and Road Initiative (BRI) as an example to examine the role of imagined intergroup contact in cognitive processing, and the representation of attention bias in cognitive content by cognitive maps across different nations.

**Method:**

This study is based on 8,348 valid cross-national questionnaires obtained. Test 1 employed structural equation modeling to analyze the role of imagined intergroup contact in the cognitive process. Test 2 utilized semantic network analysis to characterize similarities and differences in attentional allocation toward BRI-related cognitive content across nations.

**Results:**

For cognitive Process, imagined contact significantly improves stereotypes, with perceptual sensitivity and cues to action being key motivators. Perception of threat was less influential than expected. Regarding the key roles influencing cognition BRI cooperating countries and the BRICS, which combine high global and local efficiency, assume the role of cognitive ‘attraction’. Regarding the prioritization of cognitive content, attention allocation followed a power-dependent pattern—stronger nations focused on status maintenance, while weaker nations prioritized survival-related issues.

**Discussion:**

Our findings propose a dual-process framework for transnational policy cognition, combining top-down imagined contact effects and bottom-up selection of cognitive content. This study advances cognitive science by linking intergroup psychology with policy communication, offering policymakers strategies to navigate cognitive barriers in global governance.

## 1 Introduction

The development of digital media platforms and technologies has embedded the transnational communication of regional policies in an uneven global information and power space, which has not only exacerbated the adaptive and cognitive complexity of the global public but also shifted the core focus of global communication from the opinion-based “information” model to the emotional-based “cognitive construction” model (Yang and Wang, 2024). Within this context, a critical question emerges: How do transnational policies shape mutual understanding and cognition among the publics of different nations in the absence of direct contact? And in turn, how does this cognition affect the effectiveness of a policy’s transnational communication?

Indeed, the cognition and social structures of transnational groups have long posed a significant challenge to research on the effectiveness of global communication. Although “cognition” is of significant importance, research on the effects of cognition and media practices in the context of policy development from a transnational perspective remains scarce. This research gap not only constrains scholars’ cross-cultural understanding of policy communication in a globalized context, but also undermines policymakers’ capacity for precise intervention and cross-cultural adaptability in practice. Building upon this core question and the identified limitations of prior research, this paper centers on the concept of “transnational policy cognition,” which refer to the mutual understanding and cognition of policies among public across different nations. Specifically, it encompasses individuals’ perceptions and acceptance of policies, the attitudes toward establishing a trust intergovernmental relations, and the assessment of perceived benefits or threats posed by policies, all of which may ultimately influence constructive international policy interaction.

This study focuses on two dimensions—cognitive processes and cognitive content. Imagined intergroup contact affords an opportunity to examine the mechanisms underlying cognitive processing in the contexts of lacking direct interaction, whereby individuals adjust their stereotypes through mental simulation of interactions with members of other groups. However, imagined contact does not emerge spontaneously; its content is shaped by group cognitive structures. To accurately capture these structures, cognitive mapping and semantic network analysis is deployed to identify policy elements or affective cues demonstrating heightened attentional bias. Synergistically, imagined intergroup contact and cognitive map representations constitute the essential tools for measuring and understanding the “cognitive system.” Based on this, this paper conducts a cross-country empirical cognitive study taking China’s Belt and Road Initiative (Hereinafter referred to as the BRI) as a case study. Since its inception, the BRI has triggered a resource-based public opinion controversy and has gradually become a focal point for global governance and public cognition and communication. Specifically, this study will address the following four questions:

Q1: Does imagined intergroup contact mediated by the Belt and Road Initiative (BRI) significantly alter public stereotypes toward China?
Q2: (if can) What are the psychological perceived mechanisms by which BRI-related imagined contact affects stereotype development in cross-national contexts?
Q3: Which countries hold potential significance in shaping China's global cognitive system through the communication of the BRI?
Q4: What are the differences and similarities between the core and peripheral concerns regarding the BRI across different nations?

Overall, this research examines the micro-level psychological mechanisms underlying its formation through imagined intergroup interactions and semantic representations in cognitive mapping. It further reveals divergent cognitive schemas reflected in semantic network structures. This study advances comprehension of global political communication’s complexity by integrating public cognition and affective dimensions into its analytical framework. Crucially, it contributes to identifying cognitive origins of misperceptions and biases. The findings not only provide empirical evidence for exploring psychological pathways toward mutual understanding among cultural groups, but also enable policymakers to identify critical factors in policy communication and cooperation, facilitating strategic allocation of diplomatic and communication resources.

## 2 Literature review

### 2.1 Imaged intergroup contact and stereotype

Since Lippmann (2017) first proposed the concept of “stereotype,” intergroup contact has consistently been regarded as a critical factor in forming stereotypes. After that, Fiske et al. (2018) proposed the classic Stereotype Content Model (SCM) and pointed out that Warmth and Competence are the two basic latitudes that constitute group stereotypes. Within this model, warmth refers to a group’s sociability and relationship-building potential, whereas competence evaluates perceived efficacy in goal achievement. Despite stereotyping as an unavoidable way of perceiving information, intergroup contact is still one of the most effective ways of moderating stereotypes. The intergroup contact hypothesis posits that equality of status, sharing goals, and institutional support will reduce stereotypes and increase cooperative intentions, while ignorance, unfamiliarity, categorization, and competition will exacerbate stereotypes and foster division (Allport, 1954). A substantial body of research has demonstrated the effectiveness of intergroup contact in reducing intergroup prejudice, such as the stereotype-consistent contact research conducted by Zingora et al. (2021) and the meta-analysis of 713 independent samples from 515 studies by Pettigrew and Tropp (2006). However, in today’s highly segregated society, even geographically proximate groups could face community isolation or contact difficulties (Husnu and Crisp, 2010). In this context, researchers shifted their attention from socio-cultural perspectives to cognitive psychology. Crisp et al. (2010) proposed imagined intergroup contact—an indirect contact strategy involving the mental simulation of social interactions—as a means to mitigate stereotypes between groups.

‘Imagination’ can affect the public’s attitude by influencing their perceptions, as it depends on a person’s social network or some indirect contacts in their life. A large body of literature on imagined intergroup contact supports this proposition; for example, Sønderskov and Thomsen (2015) tested the effect of political party cues in contact scenarios on the effects of contextualizing intergroup contact; Moreover, intimacy relationship (Wright et al., 1997), affective choice and social cognition (Ortiz and Harwood, 2007), and self-outgroup positive trait (Stathi and Crisp, 2008) all emerged as positive factors in reducing the stereotypes of internal and external group during imagined contacts. However, most existing research focuses on imagined inter-group contact within the same cultural context, and only a few studies on racial impressions and immigrant adaptive systems (Brambilla et al., 2012) have focused on the effects of imagined contact from a transnational perspective. For example, Arcila-Calderón et al. (2022) drawing on the theoretical frameworks of intergroup contact and mediated intergroup contact—undertook a large-scale longitudinal analysis of online hate speech on Twitter to investigate mediated contact and social desirability bias across different European regions. However, social media both reveals and conceals genuine attitudes, and their study fails to address the influence of individual’s perception during contacting process. Similarly, in China, although relevant studies analyze the impact of transnational, national identity, and distance on national image (Wang and Máximo Badaró, 2023), it has not been possible to systematically test how a transnational policy or activity affects the cognitive processes and outcomes of different outgroups. This paper conceptualizes the transnational policy of the Belt and Road Initiative (BRI) as a communication medium, examining how imagined intergroup contact with it shapes cognitive trans-formations across nations globally. In addition, compared to the extensive research on the effect of imagined intergroup contact, relatively few studies have investigated the role of perceptual factors in the process of imagined contact. This study seeks to establish an integrated model in which multiple perceptual factors collectively influence stereotypes, aiming to elucidate the role of different perceptual elements in the cognitive process.

As Allport (1954) hypothesized initially, intergroup contact’s most significant positive effects occur when groups are of equal status, share the same goals, and have no intergroup competition and authority sanction. Stephan et al. (2000) have generalized this voluntary and equal-status contact into the concept of ‘quality of contact’ and have demonstrated its positive impact on stereotypes by studying group relations between Americans and Mexicans. In these studies, the perceived importance of power underlies these realist theories and practices, which influence group perceptions and choices (Fiske et al., 2018). Drawing upon these empirical findings, the present study proposes the following hypotheses:

*H1a*: Perceived Quality of Contact of the BRI in the process of imaged intergroup contact has positive effect on the stereotype of warmth.

*H1b*: Perceived Quality of Contact of the BRI in the process of imaged intergroup contact has positive effect on the stereotype of competence.

Wright et al. (1997) demonstrated that imagined contact enables the public to perceive benefits from vicarious experiences, fostering positive intergroup interactions. Turner et al. (2020) further emphasized that imagined contact reaches peak efficacy in reducing stereotypes when contact not only meets the public’s benefits but also addresses individualized needs. These benefits encompass both (a) individual affective and social identity factors (e.g., reduced intergroup anxiety and enhanced empathy) (Igartua et al., 2019) and (b) collective interests at societal and national levels, particularly the perceived significance of shared opportunities (Paolini et al., 2021). Based on these studies, this paper proposes the hypothesis:

*H2a*: Perceived Benefits of the BRI in the process of imaged intergroup contact has positive effect on the stereotype of warmth.

*H2b*: Perceived Benefits of the BRI in the process of imaged intergroup contact has positive effect on the stereotype of competence.

Perceived sensitivity refers to individuals’ beliefs about the likelihood of their exposure to a specific policy or social group. In an experiment on the investigation of predictors and drivers of intergroup contact, Kauff et al. (2021) noted that the possibility that the public can be exposed to a social policy and the social support that this policy may bring could promote positive intergroup contacts and change stereotypes. In addition to the availability of the opportunity itself (Paolini et al., 2022), Dunne (2013) posits that individuals’ personality traits—specifically their sensitivity to detect opportunities for acquiring new skills or resources from outgroup members—constitute a critical role in facilitating positive imagined contact. Based on these studies, we further hypothesize:

*H3a*: Perceived sensitivity of the BRI in the process of imaged intergroup contact has positive effect on the stereotype of warmth.

*H3b*: Perceived sensitivity of the BRI in the process of imaged intergroup contact has positive effect on the stereotype of competence.

However, in contrast to these studies, Dixon et al. (2005) argue that some scholars have been overly optimistic about the power of ‘imaged contact’. Pettigrew and Tropp (2006) have criticized that many previous studies have neglected to examine whether the contact experience is a positive or negative mental process. Spencer et al. (1999) proposed the concept of stereotype threat, which refers to the psychological phenomenon wherein an individual’s awareness of negative stereotypes about other groups creates barriers to intergroup contact and performance. Much of the research has emphasized the side effects of ‘anxiety’ (negative expectations, embarrassment, rejection, or discrimination) in imagined contact (Cakal et al., 2021). In addition to self-perceived barriers, external negative contact experiences, such as identity conflicts (Paolini et al., 2021), can further exacerbate stereotypes. According to these empirical findings, this paper hypothesizes:

*H4a*: Perceived threat of the BRI in the process of imaged intergroup contact has positive effect on the stereotype of warmth.

*H4b*: Perceived threat of the BRI in the process of imaged intergroup contact has positive effect on the stereotype of competence.

Finally, this study incorporates insights from social cognitive psychology to examine how behavioral cues may enhance users’ imagined contact intentions and contribute to stereotype reduction. External environmental triggers, media narratives, interpersonal interactions, etc., have the great potential to contribute to transforming the public’s mental imagery. For example, Capellini and Sacchi's (2021) experiments tested the effectiveness of Predictive action cues (PACs) in reducing out-group prejudice. Therefore, we assume that:

*H5a*: Behavioral cues of others towards the BRI in the process of imaged intergroup contact has positive effect on the stereotype of warmth.

*H5b*: Behavioral cues of others towards the BRI in the process of imaged intergroup contact has positive effect on the stereotype of competence.

### 2.2 Cognitive map and semantic representations

Regarding the study of cognitive content, some scholars focus on how individuals cognitively represent the relationships around them. Holsti posits that “cognition” refers to the attitudes, beliefs, and schemata that individuals form toward certain events, social groups, or even nation-states, shaped collectively by their lived contexts and experiences (Holsti, 1976). Cognitive content not only focuses on what an individual perceives but also refers to the interactive network of cognitive content arising from an individual’s perception of a policy (or an activity) (Brands, 2013). Krackhardt (1987) conceptualizes this type of social network representing cognitive representations as a Cognitive Social Structure (CSS), which seeks to uncover the latent cognitive patterns underlying social relationships. However, Cognitive Social Structure (CSS) is a micro-level framework for analyzing perceptual inaccuracies in network cognition (Krackhardt, 1987), such as discrepancies between personally perceived networks and objectively observed networks (Kilduff et al., 2008) or the influence of nodes’ positions on public behavior (Burt and Ronchi, 2007). It is not designed for large-scale cross-national or cross-ethnic comparative research. Therefore, some scholars have begun to reflect people’s understanding of the world through more macroscopic relational schemata.

Since the 1970s, cognition, as one of the core foundations of the concept of international politics, has taken an important role in intercultural communication (Kurbakova, 2015), international relations (Young, 1996), and even international conflicts and negotiations (Cheng and Brettle, 2019), the relationship between ‘belief systems’ (structure and its content) and decision-making has become a focus of scholarly attention. Holsti (1962) argued that distorted perceptions of national images, shaped by “belief systems,” may constitute a critical factor in international conflicts and communication breakdowns. This occurs because individuals’ actions and choices are fundamentally guided by their subjective cognition of how policies function within a given context. The way people search internal cognitive space is similar to the way they focus on what is going on in external physical space (Brands, 2013), which implies a commonality between the exploration of internal cognitive space and the observation of external reality space. Research on cognition in international relations primarily focuses on four analytical approaches: operational code analysis, cognitive mapping, image theory, and conceptual complexity (Young and Schafer, 1998). Among them, cognitive mapping is often used to analyze the unique relationships (e.g., causal structural networks) between image cognition in international relations (Axelrod, 2015). However, cognitive mapping still exhibits limitations in representing complex structures and in the representation of emotion and value structures; in other words, it remains inadequate for fully capturing the intricacies of human cognition. In this background, many scholars have turned to semantic network analysis for modeling the structure of cognitive content.

As the study of linguistic research shifted from the ‘semantic’ to the ‘cognitive’ (Borge-Holthoefer and Arenas, 2010) scholars have increasingly adopted semantic networks to model systems of knowledge, belief conceptual frameworks, and semantic memory structures (Kenett and Hills, 2022). Young (1996) proposed an approach to enhance cognitive mapping with a symbol-based semantic network: the WorldView. WorldView represents cognitive structures as semantic networks rather than adjacency matrices. It uses networks to characterize concepts and relationships, providing an aggregated network of textual or thematic responses (Benedek et al., 2017). In modeling cognition semantic networks, “key concepts” typically serve as network nodes, where the semantic similarity or semantic distance between lexical items defines the connective relationships among nodes in the network (Mak et al., 2021). On the one hand, scholars focus on the overall structural properties of semantic networks; Mak et al. (2021) examined the degree of centrality of concepts in semantic networks and revealed that highly central concepts can activate neighboring concepts. Additionally, Kumar et al. (2020) quantified the shortest paths between concepts by constructing binarized semantic networks. Young summarized the structural metrics of cognitive mapping into four dimensions: size, connectivity, dependency, and uniformity of salience (Young, 1996). Size, dependency, and connectivity reflect the path distances and number of relationships between concepts in the network structure. At the same time, uniformity of salience focuses on the stability of relationships within the network - higher uniformity indicates more stable existing cognitive structures and relationships.

In addition to research on network structural properties, analyzing content and hierarchical relationships within networks constitutes a crucial dimension for understanding cognitive content relationships. One of the main advantages of semantic networks is that to capture the hierarchical relationships between cognitive concepts. The node of concepts in different regional hubs or connector hubs exert different activation effects within and outside their respective modules (He, 2022). Furthermore, “concept comparison” has emerged as a prevalent analytical method among scholars (Yoon and Jetter, 2016). This approach aims to help analyze the reasons for possible changes in audience perceptions and behaviors by identifying specific domains of variation within cognitive maps. The semantic network representation of cognitive structures not only removes constraints imposed by predefined relationships but also acquires additional information regarding cognitive activation patterns and cognitive and adaptive systems. This study employs semantic network analysis and cognitive mapping to examine and compare the similarities and differences in (1) the roles assumed by the public across nations within the BRI transnational communication and (2) their core cognitive representations of the BRI initiative. This dual analytical approach provides an enhanced explanation of global audiences’ cognitive systems toward transnational policy.

## 3 Research methodology

In September 2023, this study collaborated with a prominent Chinese public opinion think tank center to administer a stratified survey themed on the 10th anniversary of the Belt and Road Initiative in 10 countries worldwide (USA, Russia, UK, Germany, Australia, Japan, Kenya, South Africa, Saudi Arabia, and Brazil). These ten countries include the Belt and Road Initiative cooperating countries, non-cooperating countries, and countries with cooperation projects but not signed agreements. To ensure sample diversity, age and educational attainment were employed as quota variables, with quota sampling implemented to mitigate selection bias. Furthermore, as subsequent analyses required cross-national comparisons of cognitive content, we utilized an equal allocation method across all ten countries. Participants were pre-screened to ensure understanding with the Belt and Road Initiative (e.g., “Have you heard of or learned about China’s Belt and Road Initiative?” “Do you understand the objectives of the BRI?” “Have you participated in any BRI–related collaborations?”), and eligibility was confirmed via platform logic checks. Ultimately, each country contributed between 770 and 850 valid responses, for a total of 8,348 cross-national questionnaires. All questionnaires demonstrated good reliability, with Cronbach’s alpha exceeding 0.7, indicating robust data quality. Based on these valid responses, descriptive and inferential statistics were generated for this study. To avoid misconceptions, the orientation of the 5-point Likert scale was applied uniformly; low scores represented negative settings, while high scores represented favorable situations. This study plans to examine how the Belt and Road Initiative, as a political communication medium, affects people’s cognitive processes and core cognitive content globally through two separate tests.

### 3.1 Test 1

#### 3.1.1 Research method

Test 1 will use Structural Equation Modeling (SEM) to examine how the Belt and Road Initiative affects stereotypes of China by influencing different perceptions of global audiences. Table 1 presents the demographic characteristics of the sample. Respondents were not informed in advance of the BRI’s identity, since a country’s designation as a partner or not is determined by official BRI cooperation agreements rather than by citizens’ subjective perceptions. Building upon this foundation, we implemented two sets of manipulation checks related to perception. First, initial screening within the questionnaire ensured participants possessed baseline knowledge of the Belt and Road Initiative (BRI) and the capacity for clear mental imagery regarding interaction with the BRI. This established the necessary foundation and clarity for the imagined contact paradigm. To examine the effect of imagined outcomes, participants were randomly assigned to either a BRI cooperation success scenario group or a failure scenario group. We then assessed the intensity of participants’ confidence in the project’s final outcome. Results indicated that the success scenario manipulation (*M* = 3.82, SD = 0.93) significantly influenced participants’ confidence in the project outcome compared to the failure scenario (*M* = 3.05, SD = 1.07) (*t* = 35.10, *p* < 0.05, Cohen’s *d* = 0.77). This outcome validates the effectiveness of the imagined outcome manipulation on participants’ perceived contact quality, benefits and perceived threats.

**Table 1:** Summary of the statistics of demographics (*n* = 8,348).

Measure	Items	Frequency	Percentage	Mean	SD
Gender	Male	4,341	52.3%	1.48	0.50
	Female	4,007	47.7%		
Age	18–28	2,939	35.2%	2.90	0.86
	29–39	3,857	46.2%		
	40–50	935	11.2%		
	51–61	617	7.4%		
Education	under senior high school	877	10.5%	1.41	0.49
	Senior high school	3,606	43.2%		
	Undergraduate and above	3,865	46.3%		

Then, the second manipulation check investigated the influence of the external environment on participants’ imagination. Participants were randomly assigned to either a scenario involving participation in a BRI conference or a scenario involving independent research on BRI materials. This aimed to gauge the extent of participants’ willingness to further understand and engage with the BRI. Results demonstrated that the conference scenario group (*M* = 4.17, SD = 0.83) significantly influenced participants’ willingness to learn about and participate in the project (*t* = 34.92, *p* < 0.05, Cohen’s *d* = 0.76), validating the role of imagined contact in shaping perceptual sensitivity and behavioral cues.

After verifying the validity of the imagination, this paper conducts a systematic confirmatory factor analysis (CFA) on seven potential variables, including the Importance of Perceived relative Power (IPP), Perceived Benefits (PB), Perceived Sensitivity (PS), Perceived Threat (PT), Behavioral Cues (BC), Warmth, Competence. Based on Confirmatory Factor Analysis (CFA), we calculated correlation coefficients to determine whether covariance or correlations exist among these factors. We also evaluated convergent validity to identify the factors constituting the measurement criteria within the model (Asparouhov and Muthén, 2009). Ultimately, we utilize Amos26.0 to construct a structural equation model (SEM) to test the hypothesized model by employing maximum likelihood estimation. This study rigorously adheres to established ethical standards and guidelines.

#### 3.1.2 Measurements

Perceived Quality of Contact: PQC refers to the perception of whether the relationship between one’s own country and the BRI is equal in status and shares the same goals. Three modified items adapted from prior research measured individuals’ feelings about the Perceived Quality of Contact toward the BRI. Respondents were asked to evaluate the perceived role of the Belt and Road Initiative (BRI) in facilitating convergent development objectives, promoting equitable consultation processes, and coordinating and mitigating international sanctions. (*M* = 2.67, SD = 0.95, Cronbach’s *α* = 0.78).

Perceived Benefits: Adapted from previous studies, four-point items were used to measure the respondents’ Perceived Benefits of the BRI. Relevant questions include: 1. The Belt and Road Initiative will bring me new job or cooperation opportunities; 2. Participating in the cooperation projects of the Belt and Road Initiative makes me feel that I am a contributing member of society (family or country); 3. ‘The Belt and Road Initiative promotes our country’s industrial upgrading and development; 4. The Belt and Road Initiative promotes cooperation with China (*M* = 2.74, SD = 0.86, Cronbach’s *α* = 0.80).

Perceived Sensitivity: Three modified items were used to measure concerns about the Perceived Sensitivity of the BRI. Respondents were required to assess the hypothetical scenario of their country engaging in Belt and Road Initiative (BRI) cooperation with China, specifically evaluating the likelihood of personal exposure to BRI cooperative projects, increased employment opportunities, and enhanced personal income levels (*M* = 2.89, SD = 0.92, Cronbach’s *α* = 0.76).

Perceived Threat: Two modified items were adapted from prior research to measure respondents’ Perceived Threat of the BRI. Respondents were asked to answer to what extent they were concerned about the potential threat of economic dependence and geopolitical expansion that the Belt and Road Initiative may bring (*M* = 3.16, SD = 1.02, Cronbach’s α = 0.79).

Behavioral Cues: For behavioral cues, we adapted three items to respond to their perceptions. First, we asked about the impact of social media and traditional official news, and then we inquired about the impact of interpersonal interactions on the BRI (*M* = 2.89, SD = 0.95, Cronbach’s α = 0.80).

Stereotypes: In terms of stereotypes, concerning warmth, we set up three questions to measure respondents’ impressions of “warmth,” including (1) Chinese people are friendly; (2) Chinese people are helpful; and (3) acceptance of being family with Chinese people. (*M* = 2.55, SD = 1.07, Cronbach’s *α* = 0.71) Regarding competence, four modified items were used to measure respondents’ cognition of competence, including accepting Chinese people as their leaders. China is a responsible country (e.g., health and environment), leading in science and technology, and Chinese people are smart (*M* = 2.88, SD = 1.13, Cronbach’s α = 0.86).

### 3.2 Test 2

#### 3.2.1 Research method

Test 2 compares the core and peripheral issues of public cognition about the BRI in different countries with the help of semantic networks and identifies the key ways to build a comprehensive image of the country. Specifically, we first assessed the consistency of respondents’ cognition regarding the seven BRI-related thematic concepts via the questionnaire. All concepts were derived from authoritative policy documents and international cooperation frameworks (e.g., official Chinese government publications, UNESCO Sustainable Development Goals). All items were phrased in neutral policy terminology (e.g., “carbon emission projects” instead of “pollution”) to preclude affective bias. Respondents were required to judge the conceptual categorization of each theme (e.g., “Does this belong to an economic theme?”). Operational definitions were employed to ensure conceptual cognitive consistency among respondents. As shown in Table 2, Cronbach’s α for all themes exceeded 0.70, indicating reliable agreement in participants’ conceptual classifications.

**Table 2:** Summary of the statistics of Seven BRI-related thematic concepts.

Measure	Items	mean	SD	Cronbach’s α
Politics	Cooperation agreement	3.84	1.12	0.87
	Regional security governance	3.74	0.98	
	Foreign policy coordination	3.68	1.05	
Economy	Trade exchanges	4.01	1.08	0.90
	Cross-border enterprises and investment	3.89	1.03	
	Employment opportunities	3.88	0.93	
Culture	Artistic performance	3.42	1.22	0.78
	Educational cooperation	3.32	0.97	
	News and media coverage	3.88	1.15	
Technology	5G Project Development	4.01	0.89	0.85
	Technology laboratory	3.78	1.02	
	Introduction of new tech	4.23	1.10	
Infrastructure	railway and highway construction	4.20	1.14	0.88
	optical cable construction	3.79	1.18	
	oil and gas pipeline	4.45	1.07	
Health and environment	Carbon emission projects	4.11	0.92	0.82
	Covid-19	3.76	0.99	
Nongovernmental contact	Travel	3.50	1.06	0.75
	Visa facilitation	4.01	1.11	
	Public welfare project	3.76	1.20	

On this basis, respondents were asked to rate their level of concern regarding seven BRI-related dimensions - politics, economics, culture, technology, infrastructure, environmental, and non-governmental contacts - on a 5-point Likert scale (1 = “highly concerned” to 5 = “not concerned at all”), based on their priorities. First, the study constructs a transnational cognitive network by treating respondents as nodes, using common interest dimension (with a score of 1) as edges, and weighting the connections by the number of jointly endorsed dimensions. (For example, if Respondent 1 and Respondent 2 both assigned a score of 1 only to the political issue, their connection is weighted as 1; if Respondent 1 and Respondent 3 both assigned a score of 1 to both political and economic issues, their connection is weighted as 2, and so on.) Next, this study categorizes different thematic concepts and examines their “core coverage” and “priority coverage” among respondents from ten countries. Core coverage refers to the proportion of respondents prioritizing a particular topic among all topics (priority coverage of a topic/total priority coverage of a topic), which focuses on the specific topic itself. Priority coverage refers to the proportion of respondents from a specific country who priorities a given topic overall respondents within this topic (number of respondents from a given country who prioritie a given topic/number of all respondents who prioritize a given topic), which focuses on the specific country’s respondents. Building on this, the study conducts a comparative analysis of the structural characteristics and conceptual relationships between the two types of semantic networks. Through network analysis, this research identifies cross-national differences in the public’s cognition of the Belt and Road Initiative at scale, revealing which pathways may play a critical role in shaping the global cognitive system regarding China’s policy.

## 4 Results and discussion

### 4.1 Structural model testing

The mean, standard deviation, and Cronbach’s alpha of all questionnaire items exceeded the recommended confidence and dimensionality criteria (Cronbach, 1949). Then, we conducted assessments of content, discriminant, and convergent validity to examine whether the model used was appropriate for testing our hypotheses. Table 3 showed the descriptive statistics of the factor loadings for each latent variable, and the measurement model containing the seven observed variables had a reasonable fit for the structural model: χ^2^/D. F = 4.2, CFI = 0.96, AGFI = 0.94, RMSEA = 0.046.

**Table 3:** Summary of measurement items (*n* = 8,348).

Latent variable	Observed variables	std.	Unstd.	S. E.	t-value	*p*	SMC	CR	AVE	R^2^_full
PQC1	PQC	0.509	1				0.26	0.73	0.48	/
PQC2		0.788	1.337	0.071	18.95	***	0.62			
PQC3		0.749	1.291	0.069	18.672	***	0.56			
PB1	PB	0.522	1				0.18	0.71	0.39	
PB2		0.634	1.622	0.12	13.576	***	0.29			/
PB3		0.637	1.531	0.111	13.731	***	0.29			
PB4		0.683	1.984	0.133	14.97	***	0.47			
PS1	PS	0.574	1				0.33	0.72	0.46	
PS2		0.664	1.27	0.062	20.329	***	0.44			/
PS3		0.698	1.564	0.075	20.741	***	0.49			
PT1	PT	0.838	1				0.70	0.83	0.71	/
PT2		0.842	1.265	0.336	3.768	***	1.02			
BC1	BC	0.661	1				0.44	0.69	0.43	/
BC2		0.635	1.147	0.069	16.664	***	0.29			
BC3		0.671	1.235	0.07	17.623	***	0.33			
C1	Competence	0.685	1				0.47	0.75	0.44	0.53
C2		0.722	0.981	0.037	26.19	***	0.52			
C3		0.695	0.978	0.039	25.272	***	0.48			
C4		0.529	0.776	0.039	20.09	***	0.28			
W1	Warmth	0.742	1				0.55	0.79	0.56	0.48
W2		0.792	1.111	0.037	30.402	***	0.63			
W3		0.713	1.014	0.037	27.216	***	0.51			

***p < 0.001.

### 4.2 Hypotheses testing

The objective of Test 1 is to examine the impact of varying perceptions in the process of imagined intergroup contact on stereotypes. Specifically, H1a and H1b tested the effects of the perceived quality of contact on stereotypes. PQC showed a significant positive effect on enhancing ‘competence’ (*β* = 0.83, *p* < 0.001), but no significant effect was found for improving cognition of ‘warmth’. Brambilla et al. (2012) summarized the stereotypes of China as an envious mix of ‘low warmth/high ability’. Fiske et al. (2018) noted that status predicts high competence, while competition predicts low enthusiasm. This suggests that the perceived quality of contact plays a positive role only in raising a country’s image of ‘international status’ but not in mitigating rivalries. In contrast, the perceived benefit positively affected changing warmth (*β* = 0.76, *p* < 0.001), but there was no significant relationship with ‘competence’. Perceived benefit can partially compensate for the competitive tendencies triggered by perceived quality of contact, which helps alleviate intergroup competition and enhances perceptions of warmth by promoting shared cooperative opportunities and mutual benefits.

As predicted by the hypothesis, H3 and H5 illustrated that perceived sensitivity and behavior cues can significantly and positively influence stereotypes, respectively. Perceived sensitivity positively affects ‘warmth’ and ‘competence’ at the 1.31 (*p* < 0.001), and 1.18 (*p* < 0.001) levels, respectively consistent with previous studies. Perceiving the possibility of contact opportunities is always a prerequisite for imagined intergroup contact (Kotzur and Wagner, 2021). Kauff et al. (2021) summarized the relevant personality variables comprising self-expansion motivation and contact confidence. At the same time, Stürmer and Benbow further categorized perceived opportunity types into six distinct classes: (1) knowledge and understanding, (2) value expression, (3) professional advancement, (4) social development, (5) personal image concerns, and (6) group image concerns (Stürmer and Benbow, 2017). According to the self-expansion model, the essence of self-expansion lies in individuals’ pursuit of diverse opportunities to enhance their sense of self-efficacy (Paolini et al., 2016). That is, individuals become more confident in imagining the potential benefits they may gain through imagined intergroup contact. Our findings showed that this self-efficacy-driven mechanism not only facilitates the enhancement of warmth-related cognitions exemplified by knowledge and understanding but also strengthens competence-related cognitions represented by professional and developmental attributes. These results significantly affect the cross-national communication of a country’s regional policies.

Behavioral cues positively affected warmth and competence at 1.50 (*p* < 0.001) and 1.21 (*p* < 0.001), respectively. Our findings further provided empirical support for the “secondary transfer effects” of imagined intergroup contact. Specifically, under conditions of benefit-driven motivation and self-expansion motivation, individuals’ direct contact experiences can positively affect secondary groups not initially involved in the imagined contact process. This effect is even more pronounced in Internet (Stathi et al., 2011). Mazziotta et al. (2015) points out that mass media influence the public’s attitudes toward imagined intergroup contact through the transmission of ‘social norms’. In addition, social media could effectively diminish the distance between ingroup and outgroup members and promote a higher sense of warmth between groups.

**Figure 1:**
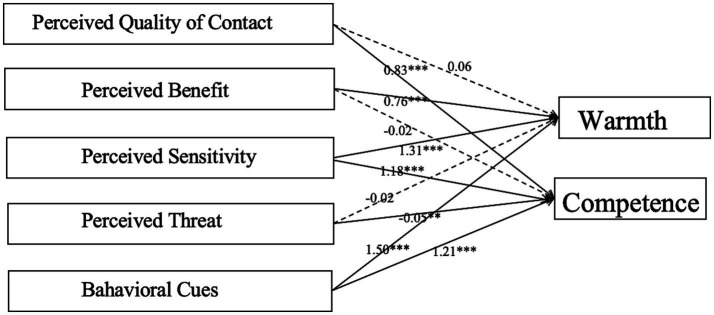
Model of TAM in imaged intergroup contact and stereotype.

For hypothesis 4, the negative impact of perceived threat was not as great as expected. There was no significant correlation between perceived threat and ‘warmth’. Although perceived threat significantly negatively affected ‘competence’, it was only at 0.05. Stephan et al. (2000) classify ‘threats’ as symbolic, real, intergroup anxiety, and negative stereotyping. Realistic threats often serve as an initial barrier to intercultural communication, while public concerns about the erosion of values, customs, or traditions serve as a more profound potential barrier to intercultural relationships and stereotypes (Figure 1).

### 4.3 Cognitive map and semantic representations

#### 4.3.1 Core hubs and connecting hubs of cognitive content

**Figure 2:**
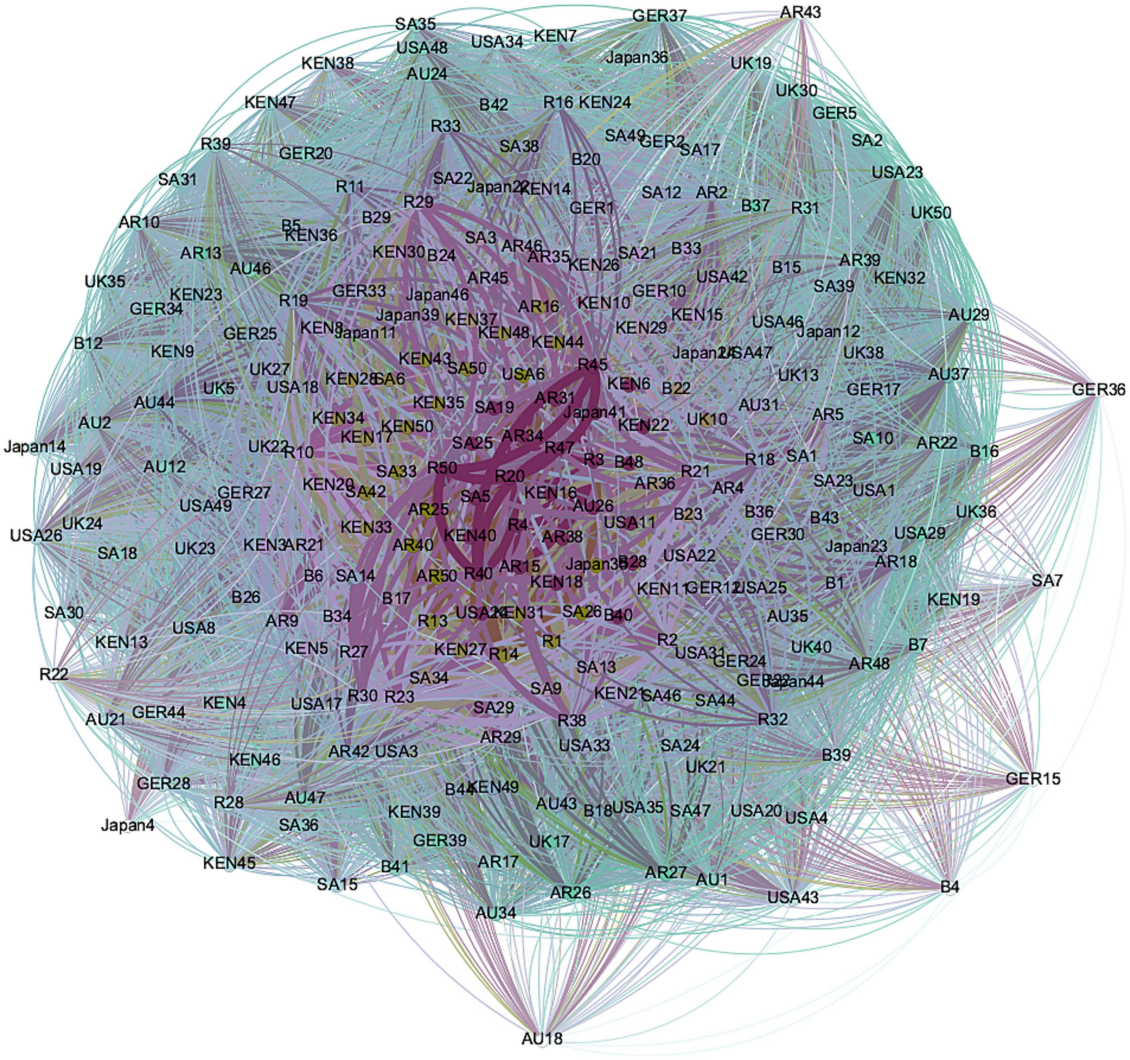
Cognition network of the BRI among ten countries’ respondents.

Next, this study will focus on cognitive content and explore the similarities and differences in the core and peripheral issues that different countries focus on. In this study, the semantic network was constructed through Ucinet for the most concerned topics of 8,348 respondents and visualized through Gephi, as shown in Figure 2. The results show that the average clustering coefficient of the nodes is 0.883, indicating a high degree of consensus and closeness between the nodes (respondents) in this cognitive network. At the same time, the network contains a total of 1,428,423 triadic closure relationships, which aligns with the high average clustering coefficient. This structural pattern indicates intense manifestations of “group cohesion” (social identity perspective) and “cognitive closure” (information processing framework) within the network (Latapy, 2008).

From the country perspective (as shown in Figure 2), the most significant number of respondents from Russia and Saudi Arabia took a center position in the network, indicating that these two countries had the largest number of respondents who expressed a great deal of concern about all seven of the topics we proposed; In comparison, Germany, the United States, Australia, and the United Kingdom have the largest marginalized respondents. The US respondents occupied peripheral positions because they gave their most concerned (score: 1) only to one type of issue, but US respondents overall demonstrated concern for a wide range of issues (political, economic, cultural, etc.). However, other peripheral respondents from Germany, Australia, and the United Kingdom have fewer common threads because they focus only on ‘culture’ and ‘non-governmental contacts’. The different reasons behind the similar positions within the network not only reflect the differences in social competencies but also reveal divergent social realities embedded within the network structure (Borgatti and Halgin, 2011). The choice of “multi-issue singularity” in the United States reflects the fragmentation and plurality of its society. The polarization of political orientations and cultural ideologies led public attention to identity-congruent and political benefit issues. In contrast, the public discussions regarding the BRI in Germany, Australia, and the United Kingdom showed a culturally-oriented perspective, emphasizing international cultural cooperation and civil society engagement.

To answer Q3, the study conducted a Modularity algorithm and betweenness centrality analysis for the nodes to reveal the hierarchical and positional relationship between nodes and subgroups. Modularity algorithm analysis can help quantify the characteristics and key roles different countries played within and outside the module (He, 2022). As shown in Table 4, the network is divided into five modules, with high-density values (e.g., 2.312, 1.568.) indicating a strong connection or interaction between the two modules. Low-density values (e.g., 0.000, 0.075.) revealed a weak or non-existent connection between the two modules. Module 3 shows the highest internal density (2.312) and mainly includes respondents from Kenya, Saudi Arabia, and Russia. This indicates that these respondents within the group share highly consistent concerns on issues and form the core group of the entire network.

**Table 4:** Module density of the Belt and Road Initiative’s cognitive network.

Modules	module1	module 2	module 3	module 4	module 5
module 1	0.000	0.000	0.000	0.000	0.000
module 2	0.000	0.726	0.785	0.380	0.075
module 3	0.000	0.785	2.312	1.568	0.489
module 4	0.000	0.380	1.568	1.590	1.017
module 5	0.000	0.075	0.489	1.017	1.000

**Figure 3:**
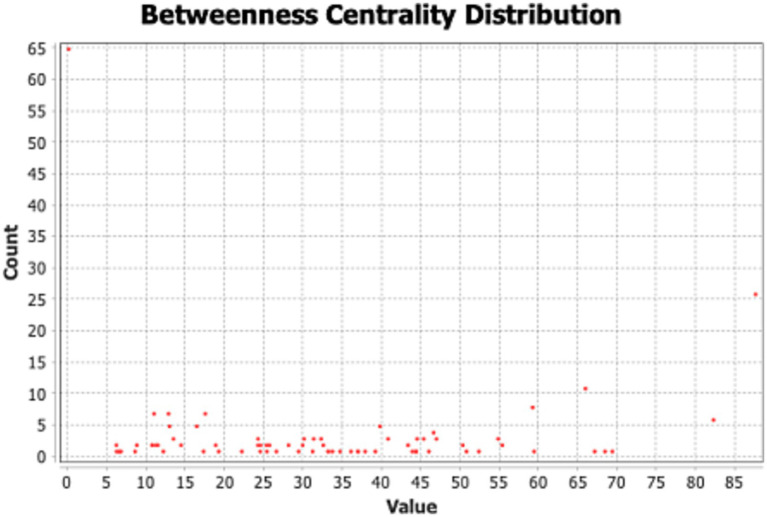
Betweenness centrality distribution of the BRI cognition network.

Regarding the betweenness centrality distribution, as shown in Figure 3, the horizontal axis (Value) represents the betweenness centrality value of nodes, and the vertical axis (Count) represents the number of nodes possessing a particular betweenness centrality value. After dividing the betweenness centrality values into three equal parts, this study regards nodes between 60 and 90 as key bridges for network links, with 46 respondents from Russia, Kenya, Saudi Arabia, and South Africa. This suggests that cooperating countries are important in connecting and attracting the common cognition of non-cooperating countries. Moreover, there is the highest density value (1.568) between Module 3 and Module 4 (As shown in Table 4), which suggests that these two groups achieve consensus more frequently in certain aspects and may share similar functions or roles (Brands, 2013). Compared to module 3, which is dominated by the BRICS countries, module 4 is dominated by respondents from South Africa (23.91%), the United States (21.74%) and Germany (17.39%), and focuses mainly on infrastructure and non-governmental contacts (score: 1, most concerned). This demonstrates that “infra-structure” and “non-governmental contacts “have emerged as key consensus points connecting new developing and developed countries regarding these two dimensions. New developing and developed countries play structurally equivalent roles within the cognitive network and exhibit a strong propensity to establish close cooperative relations.

#### 4.3.2 Core cognitive content

A semantic network is a graph-based structure representing shared cognition and the relationship between concepts. It reveals the structural relationships among words in cognition and extracts core associated concepts and their shared value emotions from the context (Kang et al., 2017). In this paper, the semantic network approach is used to represent the shared cognition system of the respondents on the Belt and Road Initiative in seven aspects.

**Figure 4:**
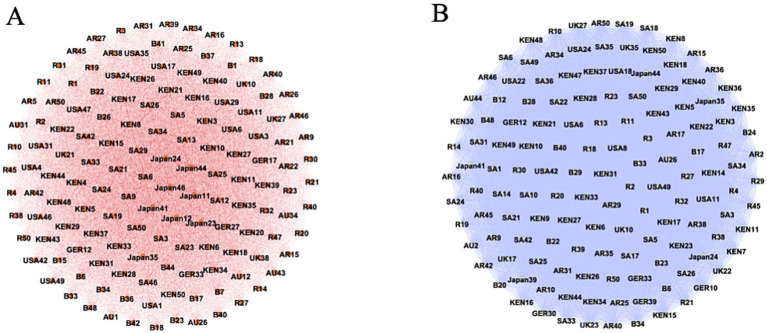
The priorities cognitive networks of global respondents on political and tech topics. **(A)** Politic; **(B)** Technology.

Among all respondents, the topic of ‘politics’ dominated the highest core coverage (22.22%), with 20.97% of priority coverage coming from Russia, 15.35% from the United States, and 13.29% from Kenya (the top three). The attention given by the public of the United States and Russia to the political implications of the BRI not only reflects their balance-of-power considerations but also highlights the increasingly geopolitical character of contemporary great-power rivalry (Zhong, 2014). A similar picture emerges in science and technology, with 31.45% priority coverage from Russia, 21.77% from the United States, and 12.90% from Kenya (the top three). Strong and weak states show different attitudes toward a transnational policy’s impact on geopolitical discourse (Song et al., 2018). States in a position of greater power are concerned with ‘protecting’ or ‘maintaining’ their existing position, as is often the case with the alternative schemes of powerful geopolitical actors (Noorali and Ahmadi, 2022). Developing countries, however, wish to gain more strategic balance through the BRI and seek new ‘living space’ in global society (Figure 4).

**Figure 5:**
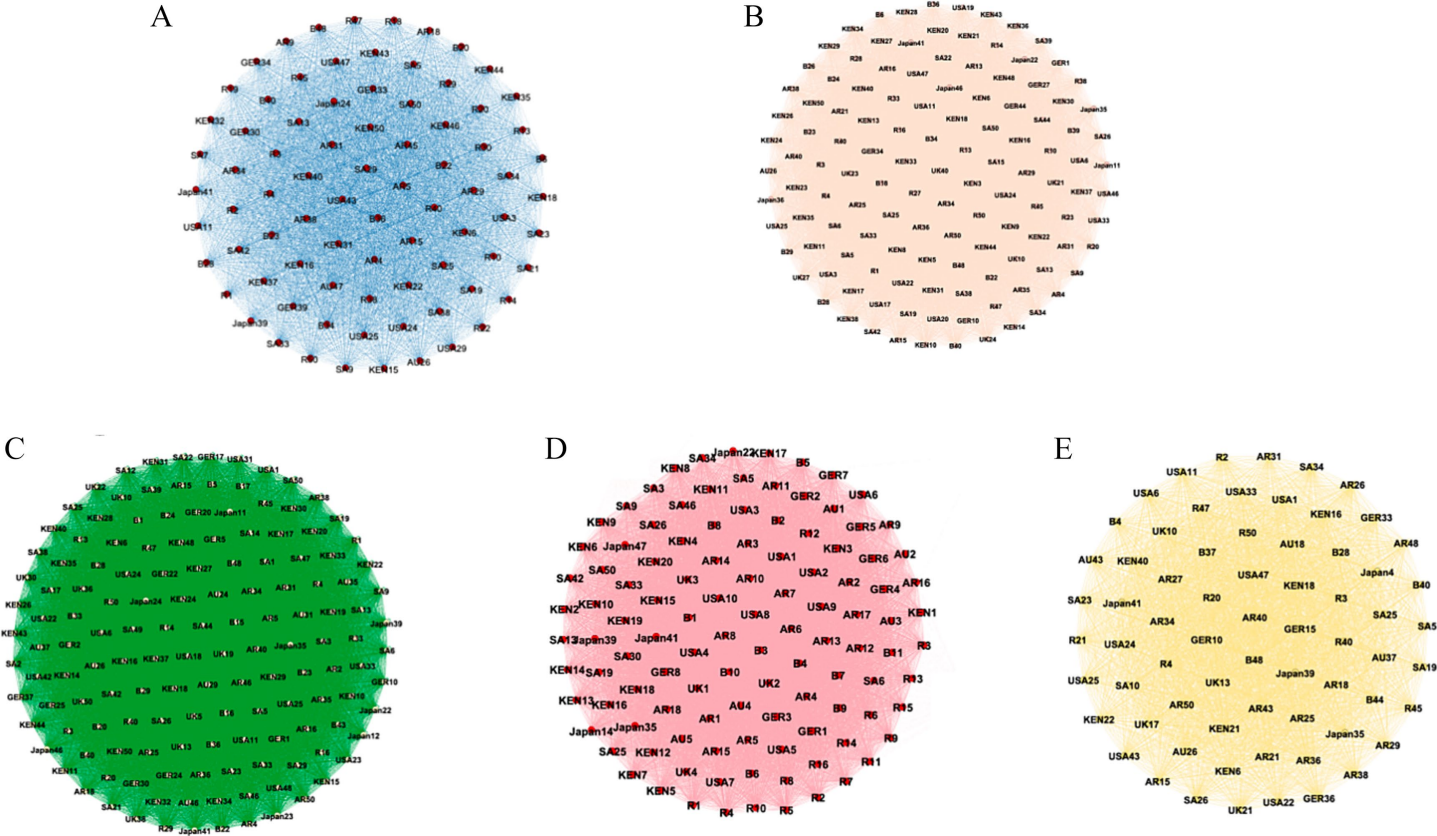
The Priorities cognitive networks of global respondents on **(A)** economy, **(B)** infrastructure, **(C)** non-government contact, **(D)** environment and health, **(E)** culture.

In addition, the study found that developed countries were more concerned with public areas such as ‘non-governmental contacts’ and ‘environment’ while developing countries prioritized ‘economy’ and ‘infrastructure’ issues, which reflects differences in the cognition content of countries with different level of power and strength (Keltner et al., 2003). High-power states tend to exhibit spontaneous social cognition characterized by affective and value-affirmative orientations, demonstrating heightened attention to security guarantees and self-concept validation issues. Regarding environmental, Germany has 21.76% priority coverage, and Australia has 17.53% priority coverage; on non-government contacts topic, the United States has 18.13% priority coverage, and Germany has 15% priority coverage. As an interregional public good, the BRI is regarded by high-powered status as a global coordination and communication medium with public attributes. In contrast, low-power countries show more sensitive needs concerns in basic survival. For example, Kenya and South Africa expressed a high level of concern about the issue of infrastructure development, with 31.45 and 14.51% priority coverage, respectively. Scholarly evidence indicates that lower-status groups exhibit a stronger propensity to accommodate existing hierarchical systems (Koski et al., 2015), attributable to their reliance on need-based and externally regulated modes of social cognition (Keltner et al., 2003). The Belt and Road Initiative (BRI) provides developing nations with an innovative adaptive mechanism for global participation, enabling them to pursue optimal resource allocation opportunities within the framework of externally regulated global cognition (Figure 5).

Finally, in the area of culture, respondents with multicultural backgrounds (e.g., Brazilians) show a stronger need and emphasis on multicultural exchanges; however, respondents in Kenya and Saudi Arabia have very low priority coverage in the area of culture (0.06 and 0.12%, respectively). The conceptual divergence rooted in religious and cultural contexts renders Chinese notions “conceptual orphans” (Koski et al., 2015) in global travel, constituting a latent barrier to the transnational communication of the BRI.

## 5 Conclusion

Overall, this study examines the Belt and Road Initiative as a case of transnational policy communication, employing structural equation modeling and semantic network analysis to investigate cognitive mechanisms in cross-cultural contexts. The research contributes to understanding cognitive(−like) behavior in interacting groups by revealing how imagined intergroup contact shapes transnational perception and how power dynamics influence collective attention allocation. One of the core objectives of political communication is to shape public cognition and understanding through the dissemination of information. Media technology developments have broken down spatial constraints on the’ contact’, and ‘imagination’ has become an important internal source in influencing political cognition (Ma, 2024). This study first examines how varying perceptions generated through imagined intergroup contact influence stereotypes in cognitive processes. The results show that perceptual sensitivity and behavioral cues play a positive role in both enthusiasm and competence. Our study extends the original propositions regarding imagined intergroup contact, demonstrating that such “imagined” contact serves as a pre-contact mechanism that fosters positive and open-minded orientations toward outgroup interactions. Furthermore, the perceived quality of contact and perceived benefits play complementary roles in stereotype modification. Perceived quality of contact reflects the public’s perceptions of a country’s international status and, therefore, has a positive effect on improving the stereotype of ‘competence’. Perceived benefits mitigate the competitive undertones associated with power perceptions and improve the stereotype of “warmth.” Finally, the negative impact of perceived threat is not as significant as we think. The perceived threat significantly negatively affects ‘competence’, but the coefficient is minimal. Public concerns about the erosion of values, customs, or traditions are potential barriers to intercultural relationships and stereotypes. Test 1 carries dual significance. First, it examines how a transnational policy can play a medium role in reducing cross-cultural stereotypes, thereby extending the theoretical model of imagined intergroup contact. Second, it would be desirable for future intervention policies to consider all perceptual dimensions to facilitate intercultural communication better.

In the Cognitive map, the BRI cooperating countries and the BRICS countries have high global and local efficiency. Specifically, members from the BRICS countries play the role of the ‘core hub’ of content in leading global cognition of the BRI. The BRI cooperating countries, on the other hand, mainly play the role of a ‘connecting hub’ for communicating (consensus) information, especially in infrastructure and non-government contacts. In addition, regarding cognitive networks’ core and peripheral content, ‘politics’ continues to be at the center of global attention. In recent years, the alternation between globalization and de-globalization has given rise to a new round of geopolitical risks and changes in the world order, and countries have been paying close attention to the regional integration strategy and the realpolitik-inspired debate behind the BRI (Petersen and Aarøe, 2013). Stronger countries pay more attention to power, security, and self-worth from the perspective of maintaining their existing positions, such as politics and technological competition, while weaker countries, by contrast, show a more sensitive need for attention to basic survival, especially infrastructure. Finally, regarding the cultural topic, the respondents with multicultural backgrounds (e.g., Brazilians) show greater attention to multicultural exchanges. With the development of social media, global citizens are becoming more active in shaping their own digital identities (e.g., “TikTok Refugees”) (Ploberger, 2017) and using these digital identities to drive cultural exchange and interaction in real life. This also lays the foundation for future global information interaction.

In summary, this study advances cognitive science by extending computational models of cognitive processes to transnational contexts through structural equation modeling and semantic network analysis of BRI-related communication. We reveal a dual-process cognitive mechanism: (1) top-down imagined intergroup contact facilitating policy cognition, and (2) bottom-up power-dependent attentional filtering shaping cross-national perception differences. These results unveil the micro-level pathways of cognitive intervention, demonstrating that individual-level cognitive processes underlying transnational policy stereotypes can be modified through specific psychological strategies (imagined contact combined with informational framing). Moreover, a key applied contribution of this study is the provision of a cost-effective toolkit for cognitive intervention in policy communication and international engagement, offering policymakers and international communicators scientific insights into how influencing micro-level cognitions (e.g., reducing intergroup anxiety and fostering empathy) can indirectly enhance policy acceptance. Finally, the research yields important implications for narrative framing in international communication, suggesting that positive, cooperative, and concrete narrative frameworks may be more effective than conflict-oriented or abstract frames in activating the psychological mechanism of “imagined contact,” thereby more powerfully shaping favorable cultural perceptions and policy impressions. However, the current study remains confined to preliminary phenomenological analysis. We also recognize that cultural background, social status, and social capital critically influence perceptions across different dimensions and the formation of stereotypes. Therefore, future research should examine the effects of more granular variables. For example, equivalent scales for political ideology and nationalism could be developed for a single country or cultural group, and the moderating or mediating roles of these variables within the information–perception–stereotype pathway could be tested. Furthermore, while this study explores the similarities and differences between global public cognitions of core and peripheral concerns regarding the BRI, what national image do these cognitive mappings reflect? How does the foundation of such a national image further propagate within complex cognitive structures? These questions require more in-depth and comprehensive analysis in future research. Overall, this study establishes a solid foundation for further research on these questions and provides a viable empirical approach for investigating the complex transnational cognitive systems involved in policy communication.

## Data Availability

The raw data supporting the conclusions of this article will be made available by the authors, without undue reservation.
